# The rheology of direct and suspended extrusion bioprinting

**DOI:** 10.1063/5.0031475

**Published:** 2021-02-04

**Authors:** Megan E. Cooke, Derek H. Rosenzweig

**Affiliations:** 1 Department of Surgery, McGill University, Montreal H3A 0G4, Canada; 2 Injury, Repair and Recovery Program, Research Institute of McGill University Health Centres, Montreal General Hospital, Montreal H3G 1A4, Canada

## Abstract

Bioprinting is a tool increasingly used in tissue engineering laboratories around the world. As an extension to classic tissue engineering, it enables high levels of control over the spatial deposition of cells, materials, and other factors. It is a field with huge promise for the production of implantable tissues and even organs, but the availability of functional bioinks is a barrier to success. Extrusion bioprinting is the most commonly used technique, where high-viscosity solutions of materials and cells are required to ensure good shape fidelity of the printed tissue construct. This is contradictory to hydrogels used in tissue engineering, which are generally of low viscosity prior to cross-linking to ensure cell viability, making them not directly translatable to bioprinting. This review provides an overview of the important rheological parameters for bioinks and methods to assess printability, as well as the effect of bioink rheology on cell viability. Developments over the last five years in bioink formulations and the use of suspended printing to overcome rheological limitations are then discussed.

## I. INTRODUCTION

**FIG. 1.**
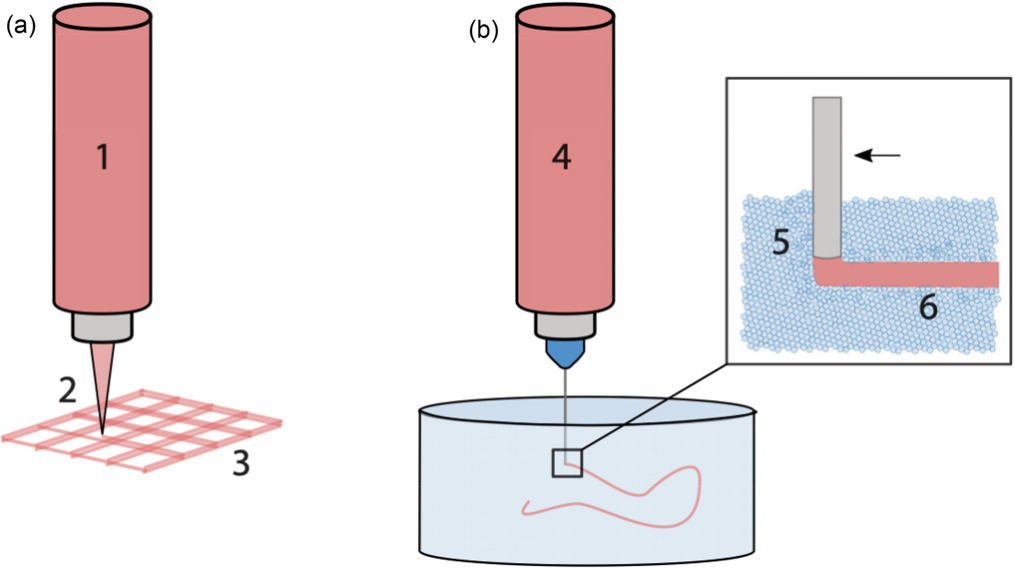
Overview of rheological requirements in a) direct (dEBP) and b) suspended (sEBP) extrusion bioprinting. In both methods of EBP, the bioink (1, 4) must be shear thinning to enable mixing with cells. During the holding time in a syringe, however, it must have some solid-like properties to prevent cells from sedimenting. During extrusion (2, 5), the bioink must again exhibit a shear-thinning viscosity profile to be forced through a small orifice. When deposited onto a flat print bed, the material must quickly recover solid-like properties, to ensure good shape fidelity and prevent the fibers from coalescing and closing the pores (3). In sEBP, the suspension medium must quickly fluidize to enable the movement of the nozzle through the media and deposition of the bioink (5). It must then very quickly recover solid-like properties to support the extruded bioink prior to cross-linking (6).

Bioprinting has developed rapidly over the past decade. What began in the early 2000s as droplet printing with modified inkjet printers has developed into a new branch of tissue engineering (TE).^1–3^ The most common method of bioprinting utilizes technology from the fused deposition modeling (FDM) technique used in additive manufacture of polymers from the filament onto a print bed with control in the x, y, and z directions.^4,5^ Extrusion bioprinting (EBP) relies on the extrusion of material through a sub-millimeter orifice, either by air and pistons or syringe-driven systems. Direct extrusion bioprinting (dEBP) describes a similar process to FDM, where material is deposited as a filament onto a flat print bed in a layer-by-layer manner as shown in Fig. 1(a).^6^ A more complex system, suspended extrusion bioprinting (sEBP), employs a suspension medium (SM) that undergoes rapid fluidization and then solidification such that it can support deposited material in 3D space prior to cross-linking, as shown in Fig. 1(b).^7–9^ Benefits of EBP include relatively low cost, good cell viability, a range of commercially available hardware and inks, and multi-material printing through the use of multiple extruders.^10,11^ The disadvantages of EBP are the time taken for printing of large constructs and, in the case of dEBP, the complex rheological requirements of materials to ensure shape fidelity while maintaining good cell viability.^12^

Ink formulations containing materials and cells are referred to as “bioinks.”^13^ These bioinks have a number of mechanical, biological, and overlapping requirements including appropriate viscosity for uniform cell encapsulation, yield stress for controlled extrusion, shear-thinning properties for extrusion through small diameter needles, viscoelasticity to protect cells from shear stresses, low thixotropy and rapid gelation for shape fidelity, hydration degree (for nutrient diffusion), and cytocompatibility for the maturation of functional tissue.^14^ These can be summarized as ensuring shape fidelity and cytocompatibility. Hydrogels traditionally cast in tissue engineering (TE) do not allow for all of these properties to be achieved. In 2013, Malda *et al.* proposed the biofabrication window as the region of moderate polymer concentration, which slightly inhibited cell survival but enabled printing of constructs with better fidelity than those that could be achieved with a lower polymer concentration.^15^ In the years since then a variety of strategies, including an array of new bioinks, have been developed to produce printed tissue constructs with excellent shape fidelity without compromising cell viability.

In this review, we first introduce the key rheological properties of bioinks and methods by which printability can be assessed. We then discuss the impact of shear stress during extrusion on cell populations, particularly their viability and phenotype, before recent methods to design advanced bioinks with appropriate rheologies. Finally, we discuss fundamentals and examples of sEBP, which overcome the limitations of bioink rheology, and how these have been implemented to introduce vascular channels in bioprinted constructs.

## II. ASSESSING RHEOLOGY AND PRINTABILITY OF BIOINKS

Injectability of a material is a commonly used parameter in tissue engineering to describe materials that can be delivered non-invasively, often carrying cargo of drugs or functional molecules.^16^ In the context of extrusion bioprinting, however, injectability does not necessarily infer printability. During bioprinting, there are a number of stages where a material must meet certain mechanical properties. Following formulation, a material must be mixed with cells to form a bioink. This bioink will then have a period of time where it is static in a cylinder or syringe prior to extrusion (holding time), during which the embedded cells must not sediment or aggregate. Next, the bioink must be forced through a small orifice without applying excessive shear, which would inhibit the viability of the suspended cells. Finally, after being deposited, the material must quickly recover some solid-like properties to support successive layers being printed, to ensure the maintenance of macropores in the scaffold, and to avoid collapsing under the force of gravity [Fig. 1(a)].^17,18^ The mechanics of the material that governs this final step are arguably the most important in dEBP and distinguish printable materials from injectable materials. In sEBP, the rheological properties of the suspension media are critical for their functions: to be displaced by a needle, allow deposition of material, and recover very quickly to support the deposited material [Fig. 1(b)].^8,19^ This section outlines the fundamental rheology of polymer solutions and common methods to assess printability of materials and bioinks.

### A. Rheology

**FIG. 2.**
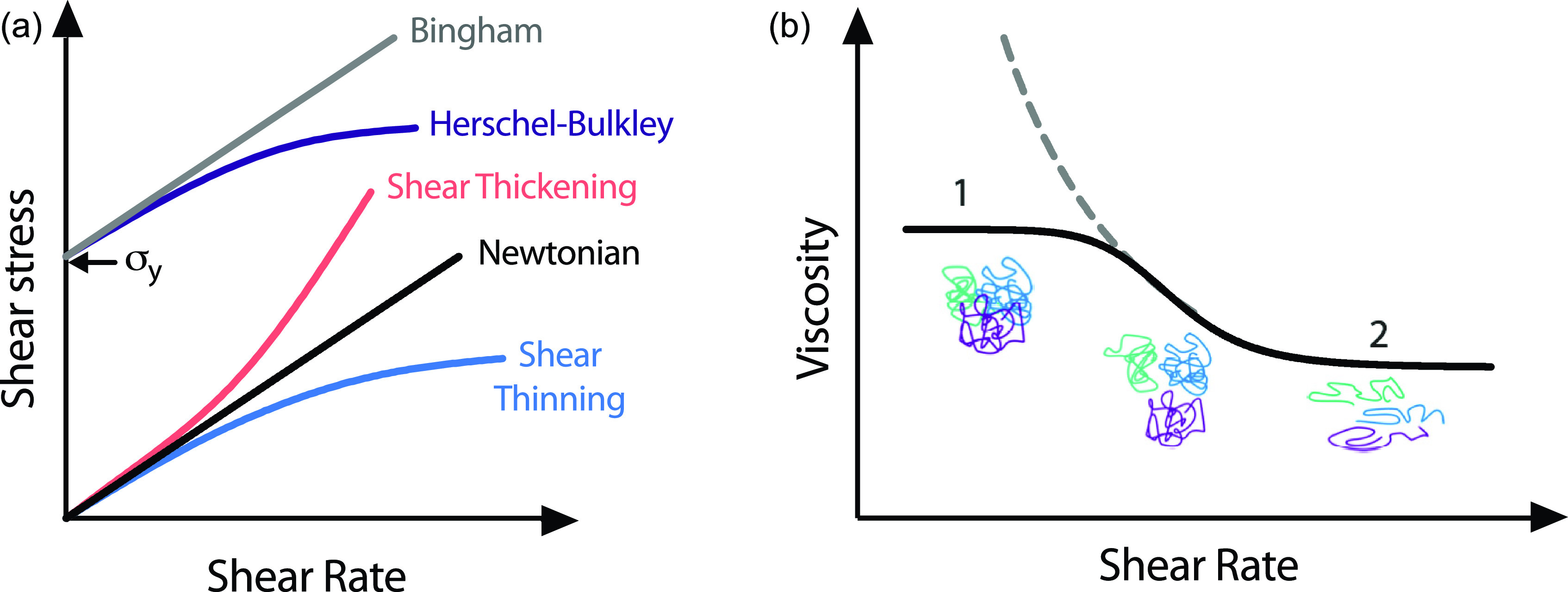
(a) Flow curves of Newtonian, shear thinning, shear thickening, Bingham, and Herschel–Bulkley flow behavior; $\sigma_{y}$ indicates the yield stress. (b) The solid line indicates the non-Newtonian shear-thinning viscosity profile between the first Newtonian plateau (1, zero shear rate plateau) and the second Newtonian plateau (2, infinite shear rate plateau). The dashed line indicates the yield stress fluid behavior, where no zero shear rate plateau is observed.

Viscosity describes a fluid's resistance to flow when a force is applied. Fluids can be classified as either Newtonian or non-Newtonian. Newtonian liquids have linear relationships with shear stress and shear rate, as shown in Fig. 2(a). Polymer solutions are usually non-Newtonian liquids, which exhibit either shear thickening or, more commonly, shear-thinning behavior [Fig. 2(b)]. These are seen as distinct changes in viscosity beyond the first Newtonian plateau (the zero shear rate plateau).^20^ Shear-thickening materials show an increase in viscosity with increasing shear stress due to the coalescence of colloids. Shear-thinning materials, however, show decreased viscosity beyond a critical shear rate, as the disentanglement and elongation of polymer chains dominate the rheological behavior [Fig. 2(b), solid line]. The second Newtonian plateau occurs when the chains cannot be further elongated, and the material reaches an infinite shear rate plateau [Fig. 2(b)].^20^ In EBP, shear-thinning behavior is critical for a bioink to be extruded through a small orifice (needle).

Yield stress materials have a critical stress (yield stress, $\sigma_{y}$) below which they behave like solids and above which they will flow.^21^ This is another important feature of bioinks, such that they will support suspended cells in a syringe, but with the application of sufficient stress (force/unit area), they will flow as liquids to be extruded in a controlled manner. Bioink yield stress behavior can commonly be fit to the Herschel–Bulkley model, as shown in Fig. 2(a) and by the dashed line in Fig. 2(b). In dEBP, increased yield stress requires higher extrusion pressures, which can negatively impact cell viability. Suspension media in sEBP are yield stress materials that typically can also be fit to the Herschel–Bulkley model, which will be discussed in Sec. V.^22,23^

Following extrusion, elastic recovery describes how a bioink recovers solid-like properties to ensure that multi-layered structures can be built up. This combination of viscous flow and elastic recovery are the constituent elements of the viscoelastic shear modulus. The shear storage (or elastic) modulus, G′, is the stored energy and describes the solid-like behavior of bioinks for elastic shape recovery or suspension of cells. The shear loss modulus, G″, is the energy dissipated by the material and describes the fluid-like behavior of bioinks that allows for cell mixing and extrusion. Under different conditions (shear rate, stress, and temperature), these moduli will often differ.

The recovery of solid-like behavior after extrusion through a needle must be fast to ensure good shape fidelity. Thixotropy describes a reversible, time-dependent decrease in viscosity as a result of a fixed shear rate or shear moduli in response to shear stress.^24–26^ The time dependency of thixotropy distinguishes it from shear-thinning behavior. With a constant shear rate, shear-thinning fluids will maintain viscosity over time, while thixotropic materials will decrease in viscosity over time. The opposite, antithixotropy (earlier termed rheopexy) will show an increase in viscosity over time with the constant shear rate.^24^ By repeatedly increasing and decreasing the shear rate or shear stress, thixotropic materials will display hysteretic behavior.^27^ Materials that are very thixotropic, in that they take a long time to recover their viscosity or shear moduli, will have limited application as bioinks to produce multi-layered structures in dEBP. Similarly, minimal or no thixotropic behavior is desirable for suspension media in sEBP.

#### 1. Polymer solutions

**FIG. 3.**
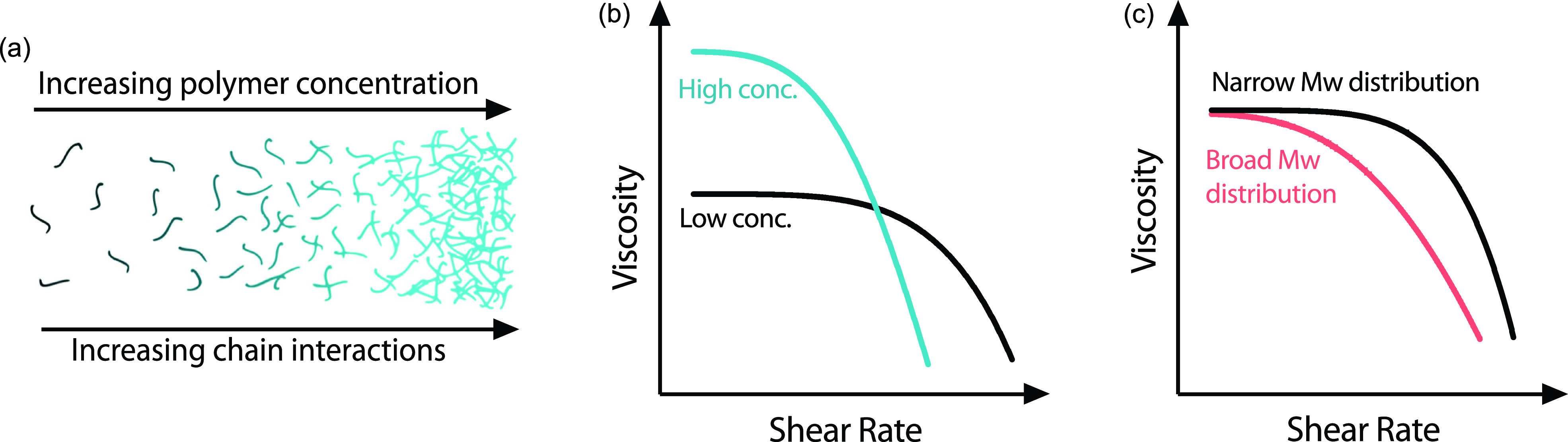
Effects of the polymer concentration on (a) chain interactions and (b) viscosity with respect to the shear rate. (c) Effects of polydispersity on shear-thinning behavior.

Beyond environmental considerations such as temperature, the shear-thinning behavior of polymer solutions is highly dependent on the polymer concentration and molecular weight distribution.^28^ Polymer solutions can be visualized as a series of chains in a solvent as shown in Fig. 3(a). In dilute solutions of low concentration, there is very little interaction between chains. With an increasing polymer concentration, the number of non-covalent interactions increases as the chains overlap. At high concentrations, chains can become entangled. The extent of entanglement is dependent on the length of the chains and the flexibility of their backbones, but increasing numbers of entanglements will always increase the viscosity of the solution.^28^

Increasing the polymer concentration causes an increase in the zero-shear rate viscosity and a reduction in the critical shear rate where shear-thinning behavior is initiated.^20^ In very highly concentrated solutions, there is typically no clear critical shear rate and just a transition to shear-thinning behavior. Increasing concentrations also causes a faster decrease in viscosity with respect to the shear rate, as shown in Fig. 3(b). The molecular weight (Mw) of the polymer, as well as range of Mw, can also affect the shear-thinning behavior. In more polydisperse solutions (broad Mw distributions), the critical shear rate is less apparent and the shear-thinning profile is less dramatic than solutions with a narrow Mw distribution [Fig. 3(c)].^29^ A range of Mw values can be introduced by using batches of the same material with different Mw values or by blending materials with heterogeneous molecular weights.^29^

### B. Determining “printability”

A number of authors have investigated key parameters that render an ink “printable,” and various methods can be used to determine the printability of a bioink, providing qualitative and quantitative outputs.^14,18,21,30–33^ The most common method to evaluate a bioink is in the printing of a multi-layered lattice/waffle/woodpile structure to determine if appropriate porosity for diffusion of nutrients can be achieved.^18^ Rheological analysis, on the other hand, can give quantitative information including the forces required for extrusion, the likely impact that this will have on cell viability and post-extrusion recovery behavior. Small changes in the bioink composition or printing conditions can be investigated rheologically both with and without cells present.^34,35^ A limitation to the assessment of printability is that, while there are some standard tests, there are no standardized experimental parameters for researchers to follow.^33,35^ Some researchers have developed mathematical models to determine printability with regard to rheological parameters and shape complexity.^36,37^

#### 1. Rheological evaluation

**FIG. 4.**
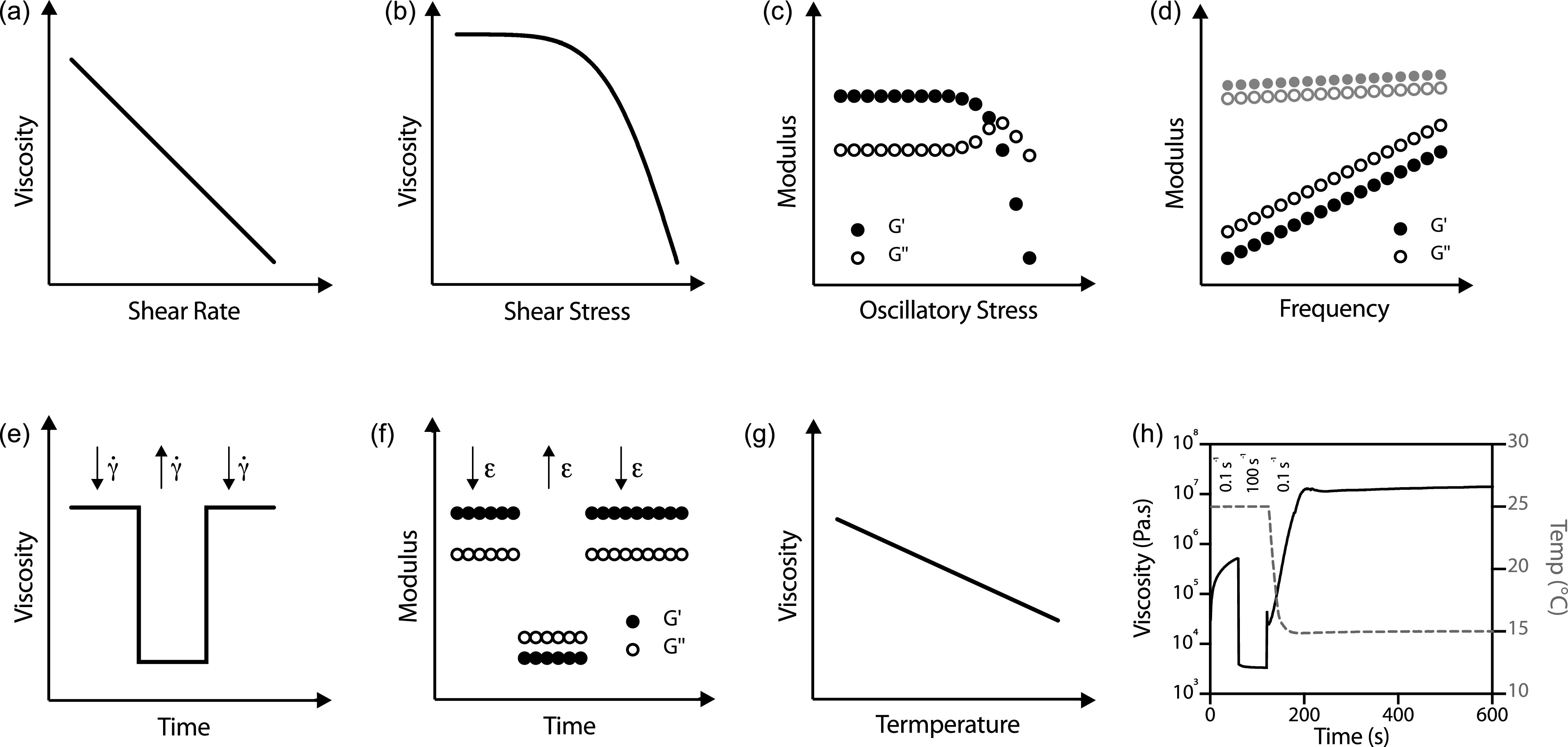
Common rheological tests for bioinks. (a) Shear rate sweep, (b) stress ramp, (c) oscillatory stress ramp (amplitude sweep), (d) frequency sweep of materials with different behaviors: solid-like gels (gray) and viscoelastic liquids (black), (e) rotational thixotropy (changing shear rate, $\dot{\gamma }$), (f) oscillatory thixotropy (changing oscillatory strain, *ϵ*), (g) rotational temperature sweep, and (h) Example of a thixotropy test to model extrusion of a gelatin–alginate bioink from a syringe held at 25 °C onto a printbed held at 15 °C. During the third (recovery) phase at a low shear rate (0.1 s^−1^), the viscosity far exceeds the original viscosity. Temperature is shown by the dashed gray line and viscosity by the black line.

Rotational (unidirectional) and small-amplitude oscillatory rheology are two techniques that can be used to assess the mechanical properties of polymer solutions. The following are some common tests performed on bioinks; typical profiles for bioinks are shown in Fig. 4, and commonly used terms are defined in Table I. For further clarification of the rheological nomenclature and symbols, the reader is referred to the Official Nomenclature of U.S. and European Societies of Rheology and NIST Guide to Rheological Nomenclature.^38,39^

**TABLE I.** Common terms in rheology.

Term	Definition
Antithixotropy	A time-dependent increase in apparent viscosity with a fixed shear rate (or shear stress)
Bingham fluid	A model of viscoplastic materials that exhibit linear shear-rate/shear stress behavior after a critical (yield) stress has been reached
Critical strain/stress/frequency	Strain/stress/frequency at which the shear moduli crossover
Herschel–Bulkley fluid	A generalized model of a non-Newtonian fluid that exhibits a non-linear strain rate/shear stress behavior after a critical (yield) stress
Linear viscoelastic region (LVR)	When the shear moduli are independent of strain amplitude
Newtonian fluid	A fluid with a linear shear rate/shear stress behavior
Non-Newtonian fluid	A fluid with a non-linear shear rate/shear stress behavior
Rheology	The study of deformation and flow of soft matter
Shear thickening	An increase in apparent viscosity with the increasing shear rate during steady shear flow
Shear thinning	A decrease in apparent viscosity with the increasing shear rate during steady shear flow
Shear loss modulus, G″	The shear modulus component representing the dissipative processes in the material
Shear storage modulus, G′	The solid-like, or elastic, component of shear modulus
Thixotropy	A time-dependent decrease in apparent viscosity with a fixed shear rate (or shear stress)
Viscoelastic liquid	A material that shows frequency-dependent shear moduli
Viscosity, *η*	The ratio of shear stress to shear rate under steady shear, the value of a liquid resistance to deformation or flow
Yield stress, $\sigma_{y}$	A critical shear stress value below which a material acts like a solid and above which a material will flow like a liquid

##### a. Shear rate sweep

It is one of the most common rheological tests for fluids and is used to investigate non-Newtonian behavior. Examples of how this can change are presented in Fig. 3. During the test, the shear rate ($\dot{\gamma }$) is gradually increased (or decreased), usually over a number of several orders of magnitudes, and the shear stress (*σ*) is recorded. From this, the apparent viscosity (*η*) can be calculated. In bioprinting, this test is very widely used to understand the flow properties of the ink during extrusion and most bioinks have shear-thinning viscosity functions as depicted in Fig. 4(a).

##### b. Stress ramp

It is a method to determine the yield stress of a non-Newtonian fluid. Above the yield stress, the applied stress disrupts the polymer chains sufficiently that the material will flow (and can be extruded).^40^ During the test, shear stress is gradually increased, while strain and strain rate are recorded for apparent viscosity to be calculated [Fig. 4(b)]. The rate of stress increase can be adjusted to model different rheological scenarios. For example, a fast increase in shear stress would better model the extrusion phase compared to a very slow increase, which could be used to model gravitational stresses (as seen in extrudate swell). This is an important consideration in designing a stress ramp experiment as the rate of stress ramping can affect the observed yield stress.^41,42^ In bioprinting, knowledge of the bioink's yield stress is important to determine the pressure required for extrusion and also the destructuring of suspension media in sEBP.^40,43^

##### c. Oscillatory stress sweeps

These are used to investigate the viscoelastic behavior of a material. The shear storage (G′) and loss (G″) moduli indicate solid-like and liquid-like behaviors, respectively, and from these moduli, complex viscosity ($\eta^{*}$, the frequency-dependent viscosity function of a viscoelastic fluid) can be calculated. The region where G′ and G″ are independent of stress is defined as the linear viscoelastic region (LVR). The following crossover point of G′ and G″ is termed “critical strain/stress,” where G″ begins to dominate, and indicates the oscillatory strain (or stress) above which the material will flow [Fig. 4(c)]. Schwab *et al.* recently proposed this test to determine both the yield point, at the end of the LVR, and the flow point at the critical stress for bioinks.^32^ This type of test is also heavily used in probing properties of suspension media for sEBP to find the critical stress at which the medium will be fluidized.

##### d. Frequency sweeps

These can be performed to determine if a bioink is acting as a viscoelastic liquid or a solid-like gel. Following an oscillatory strain sweep, a strain value that sits within the LVR is identified, and this is fixed (often 1% strain in bioinks), while the frequency is ramped during the test. In bioinks with gel-like behavior, G′ will dominate over G″, whereas the opposite is true for viscoelastic liquids. The frequency dependence is another key feature of viscoelastic liquids; with increasing frequency, both G′and G″ will increase [Fig. 4(d), black], whereas in gel-like materials, the moduli are less frequency dependent [Fig. 4(d), gray]. Gel-like bioinks typically exhibit better shape fidelity but lower cell viability.^12^

##### e. Thixotropy tests

These can be performed in both rotational and oscillatory modes [Figs. 4(e) and 4(f), respectively] to determine time-dependent behaviors with respect to both viscosity and moduli. In both cases, a common test has three steps. The first is at a low shear rate (or low oscillatory strain), the second is at a high shear rate, and the third returns to the original shear rate. Viscosity or shear moduli are measured at all times, and in the third step, the time taken to recover to the original value is of interest. In oscillatory thixotropy tests, during the low stress phases, the storage modulus, G′, dominates, and in the high stress phase, the loss modulus, G″, dominates as the material acts liquid-like, as shown in Fig. 4(f). The time taken for complete structural recovery can be very long, and so recovery to 80% or 90% of the original viscosity/moduli is often reported. In dEBP, this test is important to understand how quickly the material recovers its pre-extrusion viscosity or moduli such that it will form a stable filament.^44^ In sEBP, the thixotropic time, the time taken for the displaced material to recover, is very important to determine if the deposited material will be supported. This will be discussed further in Sec. V.

##### f. Temperature sweeps

These are useful for bioinks containing thermally sensitive materials such as gelatin or collagen. They often record viscosity or shear moduli with increasing (or decreasing) temperature [Fig. 4(g)]. They can be used to determine windows in which materials can be printed at appropriate pressures to maintain both cell viability and shape fidelity.^45^

These tests can be performed in isolation and in combination to replicate printing processes and environmental conditions. An example shown in Fig. 4(h) displays how the extrusion and solidification of a gelatin-alginate bioink can be modeled. In the print setup, the material is extruded at 25 °C at which point it acts liquid like, but the print bed is cooled to 15 °C. A thixotropy test can be designed to model the three steps of the process: (1) holding time at 25 °C in the syringe, (2) extrusion (high shear) at 25 °C, and (3) time for recovery of solid properties (low shear) on the print bed at 15 °C. By rapidly cooling the rheometer plates from 25 °C to 15 °C, the cooling of the material on the print bed is simulated.

This section has given an overview of some common rheological tests that can be performed on bioinks. There are many forces involved in extrusion of bioinks, particularly during forcing of the polymer through a needle or nozzle, and the reader is referred to a thorough review of these by Kinsella and colleagues.^46^ An ongoing limitation of rheological testing of bioinks is the lack of standardization in the parameters reported between labs. Townsend *et al.* proposed the use of the Herschel–Buckley model that relates the shear stress to yield stress, consistency index, shear rate, and flow index.^35^ This still leaves large variability associated with inconsistencies in experimental design between laboratories. In their analysis, the authors reviewed 38 studies of hydrogel precursors, 20 of which presented yield stress values, nine presented yield strain values, and nine did not present yield information. Further, values were obtained from a range of rheometer geometries and gap sizes, clearly showing the lack of standardization in rheological bioink characterization.^35^ For further information on the design of yield-stress fluids for direct printing, we refer the reader to a very detailed consideration of the topic by Nelson *et al.*^21^

In different branches of additive manufacturing, other approaches have been taken to define printability from rheological analyses, elements of which are translatable to EBP.^47,48^ M'Barki *et al.* defined a printability index for dense ceramic slurries, which considers yield stress, surface tension, and gravitational body forces in a dense, “100% infill” structure.^47^ The maximum printable height as a result of gravitational slumping was calculated using dynamic yield stress, ink density, and gravitational forces, which are all commonly reported parameters. Reduced shape fidelity in printed constructs was also shown to arise due to both gravity and surface tension where the reduction in surface energy resulted in a droplet forming instead of a cuboidal structure.^47^

#### 2. Extrudate swell

**FIG. 5.**
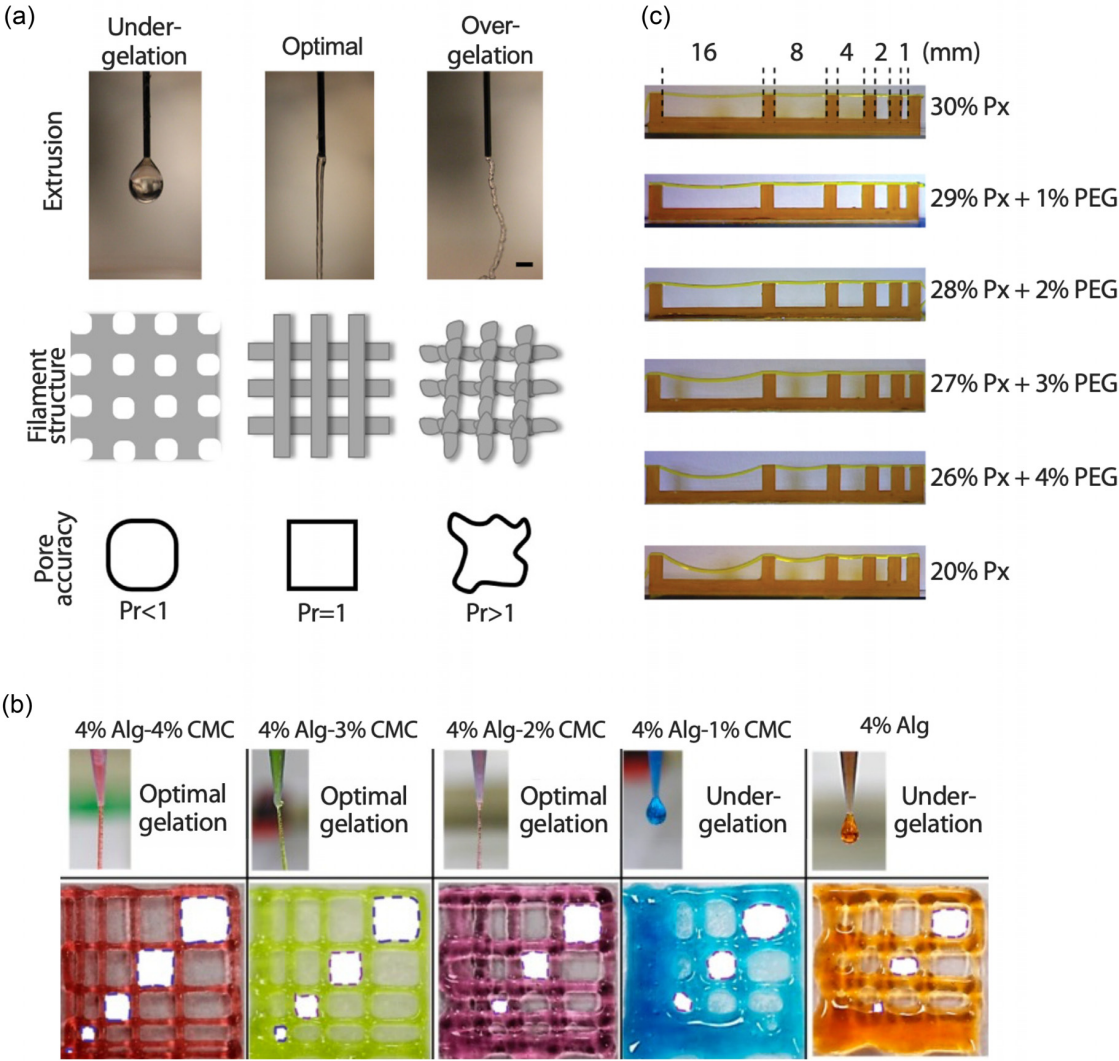
Qualitative assessments of printability. (a) Over and under gelation: extrudate swell in under-gelled or low concentration bioinks causing droplet formation, filament swelling, and pore circularity; optimal bioink formulation giving uniform filament extrusion and deposition with perfect pore geometry; over-gelation causing irregular, lumpy extrusion and filament, with unpredictable pore geometry.^57^ Reproduced with permission from Ouyang *et al.*, Biofabrication **10**, 014102 (2017). Copyright 2016 IOP Publishing. (b) Filament fusion test—at higher carboxymethyl cellulose (CMC) concentrations, there are more entanglements, and so the bioink is stiffer upon extrusion, giving rise to less filament swelling and less coalescence of printed fibers compared to lower or 0% CMC.^54^ Reproduced with permission from Habib *et al.*, Materials **11** (2018). Copyright 2018 Author(s), licensed under a Creative Commons Attribution (CC BY) License. (c) Filament collapse test—At higher poloxamer (Px) concentrations, the filament spans larger distances without deflection.^56^ Reproduced with permission from Ribeiro *et al.*, Biofabrication **8**, 035020 (2016). Copyright 2018 IOP Publishing.

Upon extrusion of a material from a needle, the ideal bioink will flow as a continuous fiber as shown in Fig. 5(a); when the extrusion is stopped, the material will stop exiting the orifice immediately. For some bioinks, a droplet is formed during or after extrusion, due to the viscoelastic nature of polymer solutions.^49^ In a needle, under shear stress, polymer chains are stretched as visualized in Fig. 2(b). Upon leaving the needle, the sudden removal of shear and drop in pressure cause relaxation of the polymer chains, known as extrudate swell, or the Barus effect.^17^ This phenomenon has been thoroughly investigated in the context of die swell in injection molding of polymers and can vary due to material, temperature, extrusion speed, and nozzle geometry.^18,50–52^ In FDM printing of polymer filaments, this problem is solved by retracting the filament. In bioprinting, this can be achieved in screw-driven and mechanical piston-driven systems (syringes); however, many commercial air-driven extrusion systems lack this option.^53^ Relaxation is also observed in filaments deposited on the print bed. Slow relaxation of polymer chains can result in thicker printed fibers than designed, leading to fibers coalescing and a reduction in shape fidelity.^54^

#### 3. Assessing shape fidelity: Filament fusion and filament collapse tests

Shape fidelity, and being able to build a porous, multi-layered 3D structure, relies on the printed fibers bridging across previously deposited layers with limited sagging or filling of the intended pore. This can be measured qualitatively by printing either a regular or decreasing pore-size lattice structure and visually inspecting to see if fibers coalesce [Fig. 5(b)].^54^ Another method to determine the maximum pore size, and also the layer height in a lattice, which can be achieved with a bioink, is using the filament collapse test proposed by Therriault *et al.*^55^ In this test, a single fiber of material is extruded over a series of pillars with increasing spacing between them [Fig. 5(c)]. The angle of deflection of the fiber at the central point in each void is then measured. The increasing polymer concentration reduces the deflection angle as the elastic modulus of the solution is increased.^56^

## III. EFFECTS OF BIOPRINTING ON CELLS (AND *VICE VERSA*)

Bioinks are, by definition, a formulation containing cells that can be processed by an automated biofabrication technology.^13^ Most commonly, these formulations also contain materials, but some studies have printed cells directly as either spheroids or organelles.^58–60^ There are many steps between taking expanded cells and a material and achieving a fully crosslinked, bioprinted structure. During this time, cells are exposed to a number of environmental stresses that can impact their viability. Shear and extensional stresses imparted on cells during extrusion are the most commonly investigated,^61^ but other steps in the process should also be considered. Further, the introduction of cells can significantly change the rheology of the material ink that they are printed in, with implications for both the possible cell seeding density and final shape fidelity.

### A. Effect of cells on bioink rheology

Complete bioink (material and cells) rheology is infrequently reported, likely due to the time and cost of expanding cell cultures to sufficient numbers to undertake comprehensive rheological assessment. This is particularly limiting when investigating primary cells, whose phenotype can be altered with long-term *in vitro* passaging. It is, however, an important consideration in the design of bioinks. Cells can be imagined as particles that constitute a volume fraction, φ, in a solvent (the material ink). There is a large volume of literature on rigid spheres in fluids, while investigations into deformable particle suspensions (that mimic cell-seeded bioinks) are fewer. Rigid spheres in suspension impede flow causing an increase in viscosity. At low particle volume fractions, the particle-particle interactions are insufficient to cause significant changes to the solvent rheology. As volume fractions increase, particle–particle interactions become more common, increasing the resistance to flow. With high volume fractions (φ > 40%), non-Newtonian shear-thinning flow behavior is commonly observed up to a high shear rate Newtonian plateau, following which some particulate systems form clusters and jammed structures, giving rise to shear-thickening profiles at very high shear rates.^62,63^

Rheological investigations into biological particle suspensions have mainly focused on blood, but there is also literature showing that fibroblasts at sufficient volume fractions can increase the viscosity and markedly change the viscoelastic properties of fluids. Two studies by Maisonneuve *et al.* investigated a range of physiologically relevant cell volume fractions on solution rheology.^64,65^ In their first study, NIH-3T3 mouse fibroblasts were prepared at φ = 20%–60% in Dulbecco's modified Eagle's medium (DMEM) with or without hyaluronic acid (HA). In DMEM, at φ = 40%, 50%, 60%, the cell suspensions exhibited a yield stress at low shear rates. As the shear stress was increased above the yield stress, shear-thinning profiles were observed. At volume fractions of 40% and 50%, a Newtonian plateau was reached at 1.7 and 4.6 Pa, respectively, before a further decrease in viscosity, suggesting that clusters of cells were disrupted beyond this plateau. The addition of HA changed the rheological profiles of the cell suspensions, as it bound to receptors on the cell surfaces. The relative viscosity ($\eta_{r}$, ratio between measured viscosity and that of the fluid without particles) was decreased with the addition of HA in all cell volume fractions, as was the yield stress and magnitude of shear-thinning behavior.^64^ In a further study, the authors investigated the effect of charged and uncharged polyethylene glycol (PEG) on the rheology of concentrated cell suspensions. At low shear stresses, the relative viscosity ($\eta_{r}$) was significantly different between aminated (PEG-NH_2_), uncharged, and carboxylated (PEG-COOH) PEG of equal molecular weight, with PEG-NH_2_ suspensions showing the highest $\eta_{r}$ and PEG-COOH the lowest across volume fractions. At intermediate shear stresses, PEG-NH_2_ was the only solution to show a critical stress at φ = 20%. At φ = 40% and 50%, PEG and PEG-NH_2_ had similar profiles, while PEG-COOH had a lower critical stress. After the decrease in viscosity, all solutions (with the exception of DMEM at φ = 60%) showed similar behavior. The observed differences at low and intermediate shear stresses are attributed to depletion effects. Uncharged PEG molecules are excluded from space between cells, changing the osmotic pressure and pushing cells closer together. This effect is counteracted by aminated PEG molecules due to interactions with the electronegative cell surface.^65^ Increased viscosity with the cell volume fraction has also been reported in low concentration alginate solutions for inkjet printing. An increased concentration of NIH-3T3 fibroblasts (1, 5, 10 x 10^6^ cells/mL) correlated with increased viscosity at a given shear rate and increased loss modulus at given angular frequency. These were all considered dilute solutions with φ< 2%, likely the reason for no observed difference in the storage modulus between cell concentrations.^66^ Diamantides *et al.* also reported an increase in low shear viscosity and storage modulus of 8 mg/mL collagen solutions upon increasing the cell density up to 1 x 10^8^ cells/mL. After gelation, however, the gels with the highest cell densities had the lowest storage moduli as cells act as weak spots in the matrix.^67^

This increase in viscosity with increasing cell density has not been commonly reported in hydrogel-based bioinks for dEBP, and the inverse has been shown. With increasing numbers of cells, the viscosity is reduced and can inhibit the formation of gelled structures.^57,68,69^ Billiet *et al.* observed that in 10 w/v % GelMA, adding 1.5 × 10^6^ cells/ml resulted in a twofold reduction in viscosity and adding 2.5 × 10^6^ cells/ml caused a fourfold reduction in viscosity when held above the gelation temperature.^70^ In other studies, a slight but not significant difference was found with 1 × 10^6^ cells/ml in gelatin–alginate blends.^57,68^ This has also been investigated *in silico* using representative volume element (RVE) modeling of spherical cells in a hydrogel and compared with *in vitro* data.^6^ This study not only used higher cell densities than that has been typically investigated in bioprinting (6.14–15 × 10^6^ cells/ml) but also found that hydrogel shear modulus was decreased with increasing cell density.^6^ Skardal *et al.* investigated the effect of three different cell types (NIH-3T3s, HepG2 C3As, and Int-407) and found that with a seeding density up to 2.5 × 10^7^ cells/ml, hydrogels formed within 20 min. When the seeding density was increased to 1 x 10^8^ cells/ml (cellular volume of 30%), the formation of the four-armed PEG hydrogel was significantly slower or inhibited in the case of Int-407 cells.^69^ As most of these cell concentrations correspond to very dilute volume fractions, the assumption based on established rheological models is that the rheological behavior would be largely unaffected. It is, therefore, likely that different mechanisms are in force. This decrease in viscosity may be a result of salts from cell culture media disrupting the polymer network through depletion effects. There could also be interference with cross-linking by sequestering of free radicals in photo-polymerisation or other reactive groups.^32^ Many more studies investigating bioinks of different chemistry, cells with ranging metabolic activity, and a wide range of cell densities are required to better understand this decrease in bioink viscosity.

The field of tissue engineering has long shown that low cell densities can be problematic for the subsequent maturation of tissue constructs as it takes longer to establish the tissue matrix of suitable mechanical strength for implantation.^71,72^ This was clearly demonstrated by Mauck *et al.* where constructs with 6 × 10^7^ cells/ml were initially mechanically inferior to those with 1 × 10^7^ cells/ml. After 8 weeks of culture, however, they were comparable, showing that with higher seeding densities, the hydrogel matrix can be remodeled faster.^73^ Achieving a balance between cell density, cell–cell interaction, cell–material interaction, and resulting viscosity presents a challenge to the field to enable immediate implantation of a tissue construct following printing.^72^

### B. Preparing for bioprinting

Mixing cells and materials is often achieved using luer-locked tubing between syringes and manually transferring material and cells between the two syringes. This ensures homogeneous mixing and reduces the formation of air bubbles compared to pipette mixing. As the syringe orifice is large, stresses exerted upon the cells are relatively low and deemed insignificant compared to the extrusion printing action.

**FIG. 6.**
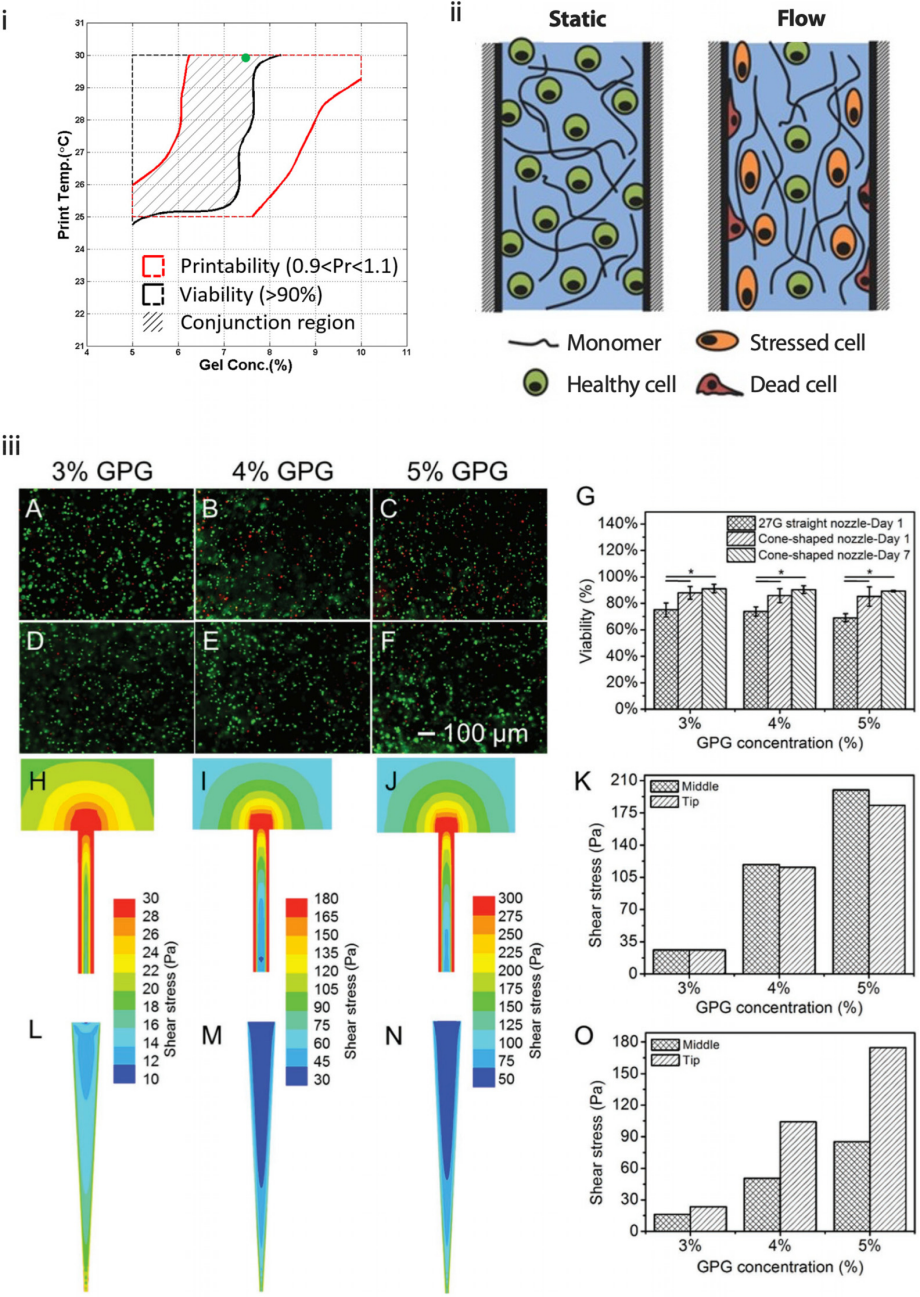
(i) Overlapping printability and viability window of increasing gelatin concentration in an alginate-gelatin bioink.^57^ Reproduced with permission Ouyang *et al.*, Biofabrication **10**, 014102 (2017). Copyright 2016 IOP Publishing. (ii) Regional variation in cell damage during flow in straight nozzles.^76^ Reproduced with permission from Blaeser *et al.*, Adv. Health. Mater. **5**, 3 (2016). Copyright 2015 John Wiley and Sons. (iii) Effects of shear stress regarding the needle or nozzle geometry on HUVEC viability in GelMA physical gels (GPGs) at 3%, 4%, and 5%. Live/dead staining and CFD-calculated shear stress regions in the 27G needle (A–C and H–J) and cone-shaped nozzle (D–F and L–N). G: quantified viability of HUVECs printed with different nozzle geometries, and K and O shear stresses at the middle and tip of straight (K) and conical (O) geometries.^80^ Reproduced with permission from Liu *et al.*, Adv. Health. Mater. **6**, 12 (2017). Copyright 2017 John Wiley and Sons.

The “holding time” refers to the period of time between cell-material mixing and extrusion; temperature is often an important variable during this phase, for example, moving materials from a room temperature cell culture hood to a heated (or cooled) insulated printhead. Zhao *et al.* found that in an alginate–gelatin bioink (held below the gelation temperature), increasing the hold time from 5 to 20 min significantly reduced viability of the A549 lung cancer cell line following printing. With a material gelation temperature of 21.5 °C, they found that viability was significantly higher when cells were held at 20 °C compared to 10 or 15 °C.^68^ By investigating the changing gelatin and alginate concentration while maintaining viscoelasticity, they also concluded that with a storage modulus between 154 and 382 Pa, the cell viability was over 90% and good print fidelity could be achieved.^68^ This group later did a more detailed study of alginate-gelatin bioinks, investigating the viability of cells held above the gelation temperature prior to printing. Using murine embryonic stem cells, they showed in three different alginate–gelatin blends, increasing the holding time again reduced viability after printing, and when held at 30 °C, viability was maintained over longer hold times.^57^ With a large increase above the gelation temperature, the temperature dependence of gelatin in the ink reduced print fidelity at low gelatin concentrations. Over a variety of gelatin concentrations, hold times, and temperatures, the authors were able to clearly show the overlapping windows of viability and printability as shown in Fig. 6(i).^57^

### C. Stresses exerted on cells during extrusion

Shear stress has long been known to play an important role in cell signaling. Changes in intracellular calcium levels in response to moderate shear stress can have large impacts on downstream signaling pathways including extracellular signal-regulated kinases (ERKs) and nitric oxidize synthase pathways. These can result in changes in the proliferative rate and differentiation.^74,75^ Excessive shear stresses disrupt the cell membrane and can induce cell death by rupturing the membrane.^76^ During extrusion, some factors that alter the shear stress experienced by cells include extrusion pressure and modality, nozzle/needle diameter, printing temperature, and polymer concentration.

Different extrusion bioprinting modalities cause varying levels of cell damage. A recent study by Ning *et al.* showed that in a number of aspects across three cell types, screw-driven bioprinting consistently induced more cell damage than pneumatic.^61^ This is likely due to the large pressure drop in the nozzle that can cause disruptions to the cell membrane, which is also seen in longer dispensing nozzles.^61,77^ Distinct stresses have also been identified in different regions of the syringe-nozzle geometry. In both conical and straight nozzles, there are shear-free extensional stresses that occur as a result of the contraction at the syringe-nozzle junction. These contribute significantly to cell death, as cells are deformed with no rotation around their central position. In the needle body, shear stresses are more prevalent, which causes not only cell deformation but also cell rotation, inducing less damage to the cell membrane.^61,78,79^ The use of straight nozzles has been shown to significantly decrease cell viability compared to conical nozzles.^70,80^ As shown in Fig. 6(iii), very high stresses [calculated using computational fluid dynamics (CFD)] are experienced in straight nozzles at the syringe-nozzle junction and continue along the walls of the straight nozzle; the lowest stresses are at the center and at the dispensing orifice. In conical nozzles, the opposite is true and stresses increase toward the dispensing orifice.^80^ With little difference in the shear stresses at the tip between the geometries, the decreased cell viability in straight needles is likely due to stresses at the syringe-nozzle junction and walls of the nozzle [Fig. 6(ii)].

Beyond the overall nozzle geometry, the diameter of the dispensing orifice has been shown in a number of studies to impact cell viability.^70,81^ Recently, Emmermacher *et al.* showed that by reducing the orifice diameter from 0.84 mm to 0.25 mm, viability of hTERT-MSCs (human telomerase reverse transcrpitase-immortalized mesenchymal stromal cells) was further reduced from 60% to 48%. In the same study, doubling the induced shear stress during printing had no significant impact on cell viability.^82^ Finally, the higher the viscosity or storage modulus of the bioink, the greater the pressures that are required to extrude it, leading to increased shear stresses. As such, increasing the polymer concentration will generally reduce the number of viable cells.^31^ In sEBP, discussed later, the viscosity of the bioink can be much lower, which enables better cell viability than dEBP.

While cell viability is a critical parameter, maintenance of the cell phenotype or pluripotency is also essential for the clinical translation of bioprinting technologies.^83^ Human mesenchymal stem cells (hMSCs) have been bioprinted from a range of sources including adipose and bone marrow and then differentiated toward chondrogenic and osteoblastic phenotypes, indicating that extrusion through a needle did not affect their ability to differentiate.^84,85^ Human-induced pluripotent stem cells (hiPSCs) and human embryonic stem cells (hESCs) have also been bioprinted using a valve-based droplet system and direct extrusion bioprinting.^86,87^ Faulkner-Jones *et al.* demonstrated that they could maintain not only very good viability but also pluripotency of hESCs and hiPSCs (by FACS) and the printing process did not induce differentiation. In appropriate culture conditions, they then differentiated both cell types into hepatocyte-like cells.^86^ hiPSCs have also been printed alongside irradiated mature chondrocytes in alginate-nanocellulose bioinks through extrusion bioprinting. Differentiation was then induced by a chondrogenic medium, and after 5 weeks of culture, collagen type II matrix production was confirmed by immunohistochemistry.^87^

### D. Crosslinking and swelling

Following extrusion, the cross-linking process may also induce cell death. Photo-cross-linking is a popular technique, initiated by the irradiation of a photoinitiator with light; for a complete review of this topic, we refer the readers to the work of Knowlton *et al.*^88^ It can be performed during or after extrusion, and there are a wide variety of photoinitiators with different absorption peaks that correspond to the wavelength of light required for cross-linking. UVA and UVB radiation (320–400 nm and 290–320 nm, respectively) have been shown to induce changes to nuclear DNA,^89^ and so researchers have focused on the use of photoinitiators that absorb near-UV or visible light wavelengths.^88^ Irgacure 2959 is a very commonly used photoinitiator at 365 nm and is cytocompatible with a number of different cell lines.^90^ Increasing the UV irradiation dose significantly reduces the viability of embedded HepG2 cell populations, however.^70^ Billiet *et al.* investigated the use of a different photoinitiator, VA-086 (absorption peak = 375 nm), and found that it gave superior cell viability compared to Irgacure 2959^70^ but gives mechanically weaker PEGDA hydrogels than Irgacure 2959 at similar concentrations.^91^ Recent work combining Irgacure 2959 and VA-086 in a dual-photoinitiator system showed very good cell viability alongside good mechanical properties in PEGDA gels.^91^ Lithium phenyl-2,4,6-trimethylbenzoylphosphinate (LAP) photoinitiators have also been used to photocrosslink methacrylated hyaluronic acid at 365 nm for 90 s where UV exposure had no effect on cell viability.^92^

Finally, as a hydrogel construct is placed in aqueous culture media for maturation, it is likely to swell with the uptake of water. In polymer solutions, there are polymer–polymer, solvent–solvent, and polymer–solvent interactions, the latter of which is described by the Flory–Huggins parameter, *χ*. This describes the interaction energy of the solvent with the polymer and indicates the solubility of a polymer in a particular solvent. The polymer-solvent interactions are critical for swelling behavior; material chemistry and the volume fraction will drastically affect water uptake.^93^ A neutral hydrogel without ionic moieties will reach an equilibrium swelling state where the thermodynamic polymer-solvent interaction and contractive force of the gel are balanced, and so the calculation of swelling behavior is relatively straightforward.^94,95^ For a hydrogel with ionic moieties, on the other hand, the ability to form ionic interactions brings about another force that must be considered in swelling, resulting in more complex thermodynamics. We refer the reader to the work of Peppas and coauthors for further discussion of these interactions.^94,96^

Swelling post-printing causes a change in the morphology of pores in a lattice structure, making them smaller, which can reduce nutrient and oxygen diffusion. This can be overcome by strongly cross-linking hydrogels but then presents new limitations in that cell migration and proliferation are restricted as the spaces between polymer chains become much smaller and hydrogels lacking dynamic interactions can become brittle.^15^ Inversely, a recent study used charge compensation between negatively charged HAMA and cationic chitosan to induce water expulsion from a printed construct. This resulted in 21% volumetric shrinkage, enabling the production of smaller features that were printable using HAMA alone. This study demonstrated that co-axial printing and charge compensation shrinkage enabled the production of tubular structures with sub-100 *μ*m inner diameters.^97^

At the whole-process scale, bioinks are often processed in the absence of cell culture media as to not impact material properties during extrusion. As a result, in the period between detaching cells from an expanded 2D culture to when they are placed in culture media for tissue maturation, the cells are starved of their normal nutrient-rich environments. Optimizing the whole process is important to maximize cell viability.

## IV. RECENT DEVELOPMENTS IN BIOINK DESIGN

The requirement for materials that maintain shape fidelity following printing and good cell viability has driven development of new biomaterials. These include chemically and physically crosslinked systems, combinations of physical and chemical cross-linking, blending materials, introducing particulates, and micro-structuring of established materials to yield new properties. This section gives an overview of some recent developments; for detailed reviews of molecular hydrogel design and cross-linking strategies, we refer the reader to further articles.^17,98^

Crosslinking strategies can be broadly divided into physical and chemical, but combinations are also used to exploit favorable properties of both.^98^ Physical cross-linking is characterized by non-covalent, reversible interactions between polymer chains. The formation of ionic bonds formed between ${\text{Ca}}^{2+}$ ions and G-groups of alginate is the most common physical cross-linking method used in bioprinting. Chemical cross-linking, however, is defined by permanent, normally irreversible covalent bonds between polymer chains. Light-driven cross-linking is very common in bioprinting with the popularity of methacrylated gelatin (GelMA) and PEGDA as bioinks due to their simplicity in manufacture, extrusion, and cross-linking. Photo-cross-linking can be initiated layer-by-layer, following embedded printing or *in situ*, using photo-permeable capillaries in place of needles.^99,100^ For a detailed review of photo-cross-linking strategies, we refer the reader to a recent review by Lim *et al.*^100^ Physical cross-linking generally results in mechanically softer hydrogels compared to chemically crosslinked hydrogels. While softer matrices enable better cell viability compared to stiffer systems, shape fidelity is limited.

### A. Dynamic bioinks

Dynamic chemistries have been introduced, which give rise to reversible bonds in materials, such that they are shear thinning under stress and self-healing when the stress is removed. Different bonds have various dissociation energies, which can be approximately translated to pressure required to extrude them. Dynamic chemistries have been used in bioinks exploiting both ionic and covalent reversible bonding and are described in detail in a recent review.^101^

#### 1. Supramolecular polymer networks and gels

Supramolecular chemistry can be described as the association of molecules through noncovalent interactions such as hydrogen bonding, transition metal complex formation, and ionic, *π*-*π*, and hydrophobic interactions.^102^ This chemistry is useful in bioprinting as these materials are sensitive to specific stimuli (mechanical, thermal, etc.) such that the interactions can be dynamically broken by applying the stimulus and reformed with its removal.^103,104^ Importantly, the binding strength of each type of noncovalent interaction is different.^102^ This allows for a range of mechanical properties to be achieved by using different or multiple interactions in designing a bioink.^105^ Several classes of supramolecular chemistry have been used in bioink formulations including guest-host complexes,^106^ supramolecular polymers,^104^ supramolecular polymer networks, and self-assembled architectures.^107^

Guest-host complexes are formed through intermolecular interactions. The host molecule is usually the larger one and often ring shaped to form multiple bonds with the guest molecule. The Burdick laboratory has led the investigations into bioprinting with guest-host complexes by conjugating *β*-cyclodextrin (cavitand host molecule) and adamantane (complementary guest molecule) to hyaluronic acid. They have used this chemistry in both bioinks for dEBP and suspension media for sEBP.^9,106^ For bioinks, studies have shown the use of the guest-host interaction alone and also the addition of methacrylate groups for UV cross-linking after extrusion.^106,108^

#### 2. Dynamic covalent crosslinking (DCvC)

Dynamic covalent bonds have an intermediate dissociation energy, lower than that of traditional covalent bonds. Therefore, DCvC polymer networks can reversibly form covalent bonds under certain conditions. The most common reactions used in dynamic covalent chemistry include disulfide exchange, boronate ester formation, aldimine formation, and reversible Diels–Alder reactions.^101^ Lee *et al.* investigated the formation of reversible imine bonds between amine-presenting silica nanoparticles and a polymeric ink based on oxidized alginate. Compared to the polymer-only ink (without aldehyde groups), the nanoparticle-containing ink (with aldehydes) had higher shear moduli and a higher critical stress. Further, by tuning the nanoparticle (SiNP) concentration, the yield stress was increased from approximately 15 Pa (0 wt. % SiNP) to around 80 Pa (2 wt. % SiNP). Print fidelity of the bioink was very good, shown by a filament collapse test and printing lattice structures with up to 30% infill without the coalescence of fibers.^109^

Dynamic coordination chemistry describes when an atom donates a pair of electrons to form a covalent bond.^110^ This was exploited to form a bioink based on bisphosphonate-modified hyaluronic acid (BP-HA), which readily forms coordinate bonds with calcium ions (${\text{Ca}}^{2+}$). This bioink showed excellent thixotropic recovery, but the hydrogel lacked robust mechanical properties post-extrusion, and so acrylamide groups were added to enable photocrosslinking upon printing. Finally, the acrylamide-modified BP-HA bioink was extruded into a suspension bath of unmodified BP-HA to achieve a multi-layered tubular structure.^111^ A cell viability in the range of 85% to 95% was maintained in all iterations, across physical (${\text{Ca}}^{2+}$), chemical (UV), and dual (${\text{Ca}}^{2+}$+ UV) cross-linking.^111^

### B. Particulate and nanocomposite bioinks

Hydrogels structured at the microscale have also been used as bioinks. Microgels of norbornene-modified hyaluronic acid (NorHA), PEGDA, and agarose were formed using a microfluidic device before photo-cross-linking or thermally induced gelation (agarose). The particle packing density was then increased through centrifugation (and removal of the aqueous supernatant) or vacuum filtration. This resulted in a jammed microgel ink where the adhesion forces between microgel particles resulted in an elastic hydrogel at low strains. The resulting inks were strongly shear thinning and had little thixotropic behavior. The ink, seeded with MC3T3 fibroblasts, was jammed by centrifugation and then extruded while maintaining the cell viability at 60%–80%.^112^

Another method of structuring was presented by Kessel *et al.* whereby microstrands of crosslinked hydrogels were produced by mechanically extruding the bulk material through a mesh. Microstrands of larger diameter (100 vs 40 *μ*m) gave rise to matrices with higher elastic and viscous moduli. Also, with longer cross-linking times, better strain recovery was observed. These materials were then printed into macroporous lattices. The printed microstrands maintained alignment over 7 days of culture and were more stable than microgels in aqueous media; increasing the aspect ratio results in more interaction between hydrogel microstrands compared to spherical microgels. C2C12 myoblasts seeded in the bulk material (which were then forced into strands) had very good viability (90%–95%) and were able to differentiate and form fused and aligned myotubes. Chondrocytes seeded outside the gel microstrands had comparable viability and formed a cartilage-like tissue matrix with a compressive modulus approximately 50% of the native tissue strength by 42 days.^113^

Nano-to-micron-sized particles have also been added to improve biological functionality or printability to bioinks. As dissused previously, the addition of particles to fluids causes an increase in viscosity as they impede flow.^62^ Laponite is a nanoclay with plate-like morphology. In aqueous media, its surfaces are negatively charged, with positively charged edges so it readily forms structured fluids. Used in combination with alginate and methylcellulose (3 wt. % each), very good shape fidelity was achieved in a lattice structure with heterogeneous distribution of the nanoclay particles.^114^ Bioactive glass, nanocrystalline hydroxyapatite, and strontium have also been introduced into bioinks with the primary aim of promoting osteogenic differentiation. They all also have beneficial effects on bioink rheology;^84,85,115^ the addition of strontium was recently shown to significantly increase the shear moduli of the GelMA precursor solution by an order of magnitude.^115^

### C. Polymer blends and additives

A number of groups have improved the printability of hydrogels with low polymer concentrations by blending them with another material. A recently published study investigated the use of methacrylated hyaluronic acid (HAMA), a hydrogel common in tissue engineering. Alone, 2.5 wt. % HAMA is of very low viscosity and shape fidelity post-extrusion is poor. In this study, it was blended with 5 wt. % gelatin and printed onto a cooled print bed (15° C) for rapid solidification of the gelatin component in order to maintain shape fidelity. The gel was then photocrosslinked such that during culture at 37° C, the HAMA maintained its shape, while the gelatin network returned to solution and was removed with culture media changes. 5 wt. % gelatin was added to a variety of methacrylated biopolymers (alginate, gelatin, chondroitin sulfate, dextran, heparin, and chitosan), and very good shape fidelity was consistently observed following irradiation with UV light.^44^

Methylcellulose and xanthan gum are popular additives to increase the viscosity of bioinks.^54,114,116^ Rastin *et al.* showed a doubling in viscosity with the addition of 8 wt. % methylcellulose (MC) to 5 wt. % GelMA. They also observed a reduction in extrudate swell; in GelMA alone, a droplet was formed at the nozzle tip, whereas MC only (and MC/GelMA combinations) produced optimally shaped fibers.^116^

## V. OVERCOMING RHEOLOGICAL LIMITATIONS WITH SUSPENDED BIOPRINTING

Section IV described developments in bioink design to alter the rheological properties of the ink for dEBP. A number of groups have circumvented the issue by, instead of changing the ink, changing the print bed. Precisely, they have transitioned from a 2D print bed to a 3D print bath containing a suspension medium (SM) that acts to support the extruded bioink prior to stabilization of the final structure by cross-linking.^19^ At rest and below its yield stress, a suspension medium exhibits solid-like properties. Upon application of stress that exceeds the yield stress such as movement of a needle and deposition of a bioink, the media are fluid-like, flow, and can be displaced. Following removal of the applied stress, the suspension media very quickly recover their solid-like properties in a “self-healing” manner, entrapping and supporting the deposited bioink prior to cross-linking.^117^

This approach also referred to as freeform, embedded, and gel-in-gel printing allows for omnidirectional printing, without the limitations of overhangs, build direction, internal voids, and irregular scaffold geometry. Most importantly, in the context of this review, bioinks of very low viscosity such as collagen solutions can be printed into complex geometries using this technique. The earliest example of this approach was by the Lewis group in 2011,^118^ and a number of methods have been developed since. These include chopped slurries,^8,119,120^ fluid gels,^7,121–124^ nanoclays,^125,126^ microgels,^117,127,128^ polymer networks with dynamic or reversible bonds^9,111^ and viscous solutions.^129,130^ The increased shape complexity that can be achieved using this technique has made it an effective technique to print vascular networks within a tissue construct.^118,119,131^

### A. The rheology of suspension media

The fundamental principle of a successful suspension medium is that of a Bingham plastic: a self-healing yield stress material with minimal thixotropic behavior. The material must recover its original viscosity or shear moduli very quickly following deformation to support extruded material. Maintaining shape fidelity when printing into a suspension medium is challenging as its properties must be compliant with the inertia of the needle and extruded bioink. This includes ensuring accurate deposition of the bioink and how fluidization of the media affects already deposited material. Many of the mechanisms by which imperfections appear and resolution is controlled in sEBP have been investigated by the Angelini laboratory with microgel (Carbopol) suspension media.^127,132,133^

Interfacial instability is seen when deposited material breaks up into non-continuous filaments. This occurs when at small length scales, the capillary pressure at the interface of the suspension medium and bioink is higher than the yield stress of the suspension medium, as shown in toroid formation.^107,134,135^ It was, however, favorably exploited to enable embedded droplet printing by Nelson *et al.*^117^ Interfacial tension is often negligible when both a bioink and SM are aqueous, which is common to maintain cytocompatibility of both systems, and so the yield stress and elastic behavior of the SM determine the achievable feature resolution.^125^ Increasing the SM yield stress has been shown to enable the production of smaller printed features.^136^

A number of different filament morphologies were reported by Jin *et al.* when printing a gelatin-alginate bioink into a nanoclay (Laponite) suspension bath. At low alginate concentrations, the filament had a larger diameter and rougher surface finish, but with increasing concentrations, the filament became thinner.^125^ This concentration effect reflects findings of Senior *et al.*, whereby using dyes of differing molecular weights, diffusion into a suspension media was reduced as the molecular weight was increased.^122^ At low nanoclay concentrations (0.5%, $\sigma_{y}$ = 0.001 5 Pa), Jin *et al.* observed that the filament had a very rough surface, likely due to low interfacial tension between the ink and suspension media. With increasing nanoclay concentrations, the storage modulus of the SM was increased and the filament was more regularly circular in the cross section although extrudate swell was reported. At very high concentrations (8% nanoclay, $\sigma_{y}$ = 15.78 Pa), the sides of the filament were compressed into a rectangular cross section due to the higher yield stress of the suspension media.^125^

During high-speed printing into a suspension medium, it has been shown that an air gap can form between the moving needle and the surface of the suspension bath. This, in turn, can cause recirculating instability in the printed material.^133^ This was found when the material was extruded at 1 m/s, a rate that would likely have a significant impact on cell survival. While unlikely to be used in the production of a tissue construct, the rheological findings are interesting.

As the suspension media are fluidized, there is potential for this to affect already deposited regions of the scaffold, particularly as the suspension media are displaced by newly deposited material. To this extent, the bottom-up approach is likely necessary or, as O'Bryan and colleagues have alluded to, path planning algorithms are required to limit disruption to previously deposited material if uncrosslinked. Alternatively, based on the thixotropic time or gelation time, it should be calculated when regions can be revisited to move through or extrude more material.^132^ A simple approach to counteract this is to minimize the volume of media fluidized by balancing print speed and extrusion pressure. This is particularly important in printing very small features such as vascular networks and is unique to the rheological properties of each suspension medium.

Grosskopf *et al.* investigated how tunable features including the matrix composition, print path, speed, and orifice diameter affect the locally yielded region in a polydimethylsiloxane (PDMS) SM. Using fluorescent particles in the SM, they used particle image velocimetry to investigate matrix deformation around the moving nozzle and the Oldroyd number was used to characterize the size of the yielded area. The velocity flow fields reduced in size as the yield stress of the material increased due to the increased PDMS content. The matrix with the highest PDMS content had the least thixotropic behavior (fastest elastic recovery) and resulted in the best print fidelity of the three PDMS concentrations investigated.^22^ As mentioned, Nelson *et al.* investigated oil-in-water embedded droplet printing and, with the well-established rheological properties of aqueous carbopol, defined a scaling curve of the droplet diameter and needle translation with a constant flow rate of the mineral oil ink phase.^117^

### B. Printing into viscous fluids

Shear-thinning viscous fluids have been used as suspension materials to print into. As mentioned, the earliest example of suspended bioprinting was by the Lewis group in 2011. They produced 3D microvascular networks using a Pluronic F127 suspension medium, modified with diacrylate groups to enable photocrosslinking. The ink was also Pluronic F127, used slightly above the critical micelle concentration such that it possessed a shear-thinning viscosity function but maintained filament-like morphology on extrusion. Directly following extrusion, aqueous acrylate-modified Pluronic F127 (layered on top) filled the void left by the needle in the suspension medium.^118^ Since this study, shear-thinning hydrogels have been designed as suspension media that do not require a filler layer as they are very strongly self-healing following stresses associated with needle movement and bioink extrusion.

Guest-host hydrogels are very strongly shear thinning and have been used as suspension media. A study by Highley *et al.* demonstrated the use of adamantane and cyclodextrin-modified hyaluronic acid as both suspension media and bioink. They were able to co-print multiple cell-seeded bioinks without the limitation of the build direction or geometry. Further, with the addition of methacrylate groups, they produced perfusable channels within bulk hydrogels.^9^ A more recent study used xanthan gum to produce freeform printed truncated tubular structures and a cell-seeded methacrylated xanthan gum bulk hydrogel with perfusable channels.^130^ The bioink used in sEBP can also be photocrosslinkable, as seen in an acrylamide-bisphosphonate-hyaluronic acid bioink that was extruded into a bisphosphonate-hyaluronic acid suspension medium.^111^

### C. Particulate suspension media

Often referred to as gel-in-gel printing, these methods use hydrogel microparticles as a suspension medium. They can broadly be classified as microgels, but the method of particle production often differs, which has implications for the particle size, size distribution, and particle morphology. These factors all affect the rheology of the suspension media. Most importantly, these parameters affect the yield stress and thixotropic behavior of the suspension media and the time taken for the SM to restructure to its original solid-like state. These directly impact the resolution and shape fidelity of the printed construct. An overview of some strategies is shown in Table II.

**TABLE II.** Methods for printing particulate gel-in-gel suspended extrusion bioprinting.

Suspension method	Material	Particle size	Morphology	References
Microgel	Carbopol	<10 *μ*m	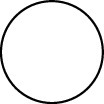	43, 127, and 137
Fluid gel	Agarose	50 (20–110) *μ*m	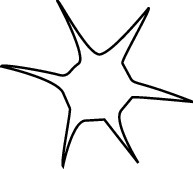	122, 123
	Gellan gum	20 (20–80) *μ*m	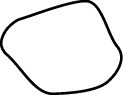	124
Chopped slurry	Gelatin	30 (10–500) *μ*m	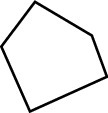	8, 139
	Alginate	30 (10–500) *μ*m	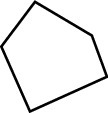	119
	Gelatin + gellan gum	60–460 + 50 ± 32 *μ*m	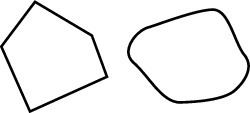	137
Cooled slurry	Gelatin	20 (10–30) *μ*m	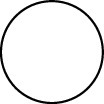	128
Organoids	Pluripotent stem cells	20 (10–30) *μ*m	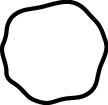	131
Nanoclay	Laponite	1 × 25 nm	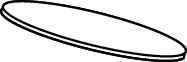	125

Carbopol is a broad term for a range of commercially available microgels based on polyacrylic acid. Carbopol materials undergo a sol-gel transition in aqueous solutions above pH 5.5, which deprotonates carboxylic acid groups in the polymer chains. This causes electrostatic repulsion resulting in swelling of the microgels to produce granular (diameter <7 *μ*m) suspension media. At high carbopol concentrations, the pH of the granular medium has significant effects on its yield stress and shear moduli, which, in turn, have a significant impact on print resolution.^137^ Different carbopol materials have been used as suspension media to produce tissue constructs and very high-resolution acellular constructs.^8,127,136,137^ Silica nanoparticle microgels have also been investigated as suspension media for high fidelity sEBP and extruded droplet printing.^22,117^

Fluid gels are a class of microgels whose particles are solidified under shear, resulting in particles of irregular morphology and varying sizes depending on the polymer used, mode of gelation, and shear rate applied during gelation. The first use of fluid gels for embedded printing was demonstrated by Moxon *et al.* in printing an osteochondral plug seeded with human chondrocytes and osteoblasts in defined regions.^7^ The fluid gel matrix was composed of agarose particles with a “hairy” morphology and has been used since in further studies.^121–123^ The hairy or dendritic morphology, shown in Table II, gives both short- and long-range interactions between particles and has been shown to give comparable or faster recovery of viscosity than jammed slurries formed by chopping crosslinked gels.^122^ Gellan gum has also been used as suspension media by Compaan and colleagues.^124^ Gellan gum is weakly thermo-gelling and strongly ionically crosslinked, and so a combination of cross-linking methods can be used to form fluid gels.^138^

Chopped particles, such as the gelatin slurry first produced and now commercialized by the Feinberg lab (Freeform Reversible Embedding of Suspended Hydrogels, FRESH) contain CaCl_2_ to allow cross-linking of materials during printing.^8^ The first iteration of this technique (FRESH v1.0) formed a slurry by chopping cooled gelatin, and the second (FRESH v2.0) introduced stirring during cooling from 45^∘^C to room temperature, similar to the formation of thermally cross-linking fluid gels.^8,128^ In both iterations, the particles were centrifuged to produce a jammed material with high packing density. The second iteration drastically reduced polydispersity of the particles to produce smaller, more uniform microgel particles. As a result, the authors showed drastically improved print resolution of collagen solutions compared to the first iteration (20 *μ*m compared to 250 *μ*m).^128^ In both these techniques, heating the suspension medium to 37° C liquefies it releasing the printed structure. This technique has been adopted by a number of labs around the world and was recently used to print a model of the cardiac ventricle. A collagen ink was used to form outer and inner walls that had a cell-only ink of human stem-cell derived cardiomyocytes and cardiac fibroblasts printed between them. After 7 days, cells became striated and interconnected and were able to spontaneously contract, shown by calcium imaging of the printed constructs.^128^

**FIG. 7.**
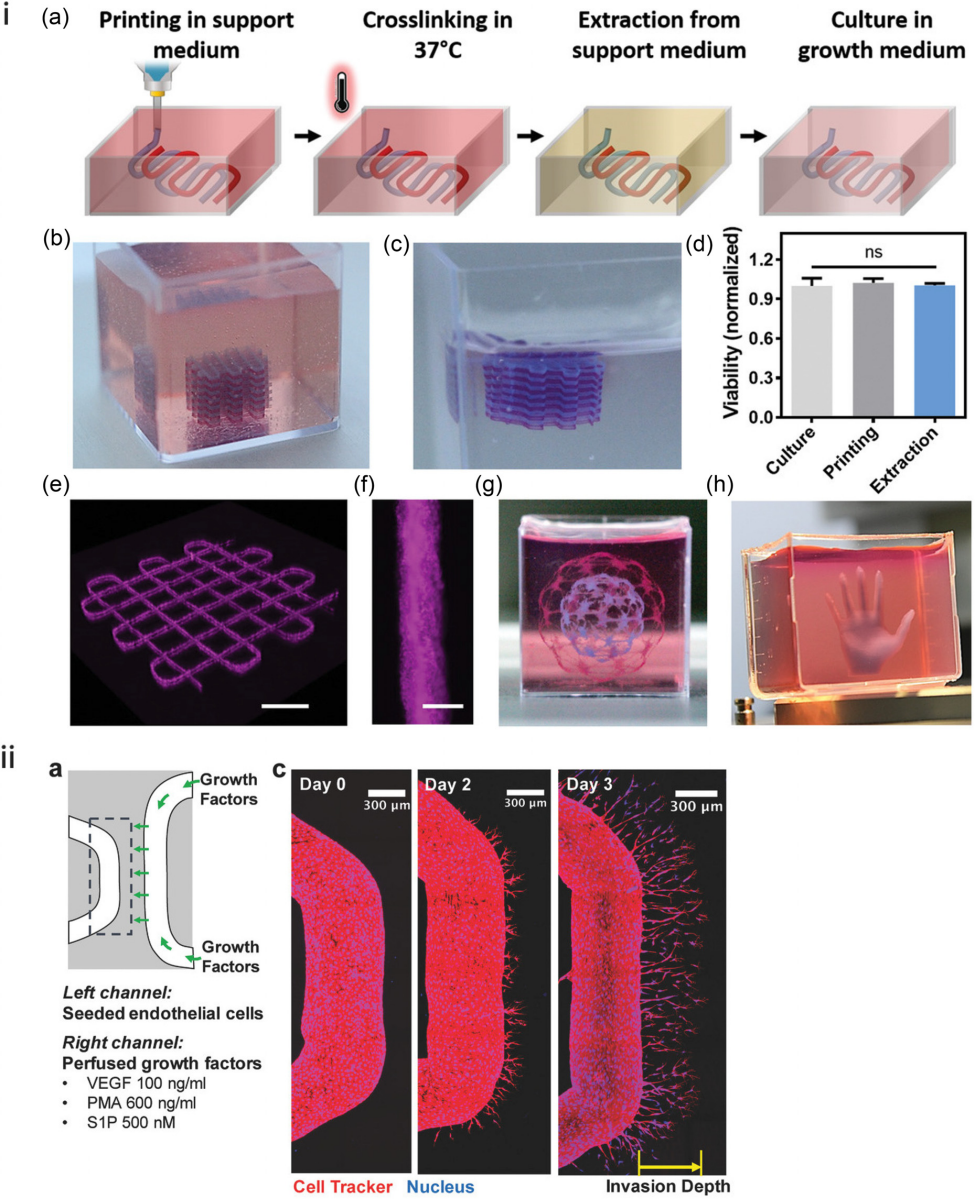
Examples of suspended printing: (i) (a) extrusion, cross-linking, retrieval, and culture of omentum hydrogel bioink in an alginate/xanthan gum support bath. (b)–(h) Examples of printable geometries; (d) cell viability during printing and extraction is unchanged.^119^ Reproduced with permission from Noor *et al.*, Adv. Sci. **6**, 11 (2019). Copyright 2019 Author(s), licensed under a Creative Commons Attribution (CC BY) License. (ii) Printing of tubular structures in guest-host hydrogels: (a) schematic and (c) temporal formation of vascular sprouting toward the growth factor channel.^142^ Reproduced with permission from Song *et al.*, Adv. Funct. Mater. **28**, 31 (2018). Copyright 2018 John Wiley and Sons.

A similar approach was taken by Noor *et al.* in the production of thick perfusable cardiac tissues. They produced a slurry of alginate/xanthan gum microparticles by a similar method to Hinton *et al.* and used a personalized bioink containing omentum gel alongside a sacrificial gelatin ink for the production of vessels. Heating the printed construct in its suspension media to 37° C for 45 min allowed the extruded structure to cross-link and the gelatin to liquefy before the alginate suspension media were aspirated and replaced with culture media [Fig. 7(i)].^119^

In dEBP, gelation must occur fairly quickly to prevent the structure from collapsing. In sEBP, there is a larger time window for this to occur, but the continuous phase between gel microparticles (or the particles themselves) can be functionalized to initiate cross-linking of the extruded filaments directly following printing.^8,139^ Divalent cations (CaCl_2_) are often introduced due to the popularity of alginate as a bioink. A recent study used oxidized alginate as the continuous phase, which bound to a carbohydrazide (CDH)-modified gelatin bioink to form a crosslinked structure.^139^ This can be beneficial as the printed part begins to solidify immediately upon extrusion into the suspension media but may result in lack of fusion between subsequent layers or even solidification of material in the needle prior to extrusion if the concentration of the cross-linking agent is too high. This can cause irregular filament deposition, similar to the observations of Ouyang *et al.* in over-gelled deposition in dEBP [Fig. 5(a)].^57^

### D. Printing vascular networks

One of the currently unmet challenges in tissue engineering is introducing a vascular network to a large tissue construct. Vessel formation is common as part of the inflammatory response to a foreign body upon implantation of an engineered tissue construct.^140^ In the case of large tissue constructs, however, the rate of vessel growth is not sufficient to prevent a necrotic core from forming in the engineered tissue. Suspended printing enables the printing of tubular structures and vascular networks as the limitations over geometry and bottom-up fabrication are removed.

A common strategy is to print a vascular channel from a sacrificial material in suspension media, cross-link the media, and then flush the sacrificial material leaving a tubular void.^119,126,141–143^ Compaan and colleagues used a mixture of gelatin and gellan chopped microgels within a gelatin continuous phase to print vascular structures using sacrificial 2% alginate before cross-linking the continuous gelatin phase using transglutaminase (TG).^144^ As soon as TG was added (prior to printing the sacrificial alginate structure), the gelatin began to cross-link giving a limited print window, and this study reported large changes in the aspect ratio of the printed filament 30 minutes after adding TG.^144^

Two tubular structures were printed in the study by Song *et al.* where guest-host chemistry was used to produce both the suspension media and the sacrificial ink. The suspension media, composed of adamantane- and norbornene-modified hyaluronic acid (HA) along with cyclodextrin-modified HA, provided a viscous fluid hydrogel bath, which, following the extrusion of a sacrificial ink, was crosslinked by a thiol-ene reaction between a di-thiol crosslinker and the norbornene groups. In one “vessel,” endothelial cells (HUVECs) were seeded and the other vessel was used to perfuse angiogenic growth factors (VEGF, PMA, and S1P). The release of factors into the protease degradable support hydrogel allowed directional sprouting of endothelial cells toward the channel with growth factors. Over 3 days, endothelial cells were shown to invade the central region with branches up to around 400 *μ*m in length as shown in Fig. 7(ii).^142^

In the Lewis group, formation of vessels has been taken one step further. Their technology of “sacrificial writing into functional tissue” (SWIFT) replaced the suspension media with hundreds of thousands of cell spheroids, with a collagen/matrigel continuous phase, through which a vascular network was printed using a sacrificial hydrogel.^131^ The resulting structures are one of the closest resemblances to tissue that has been produced in bioprinting; the cell density is very high, closely mimicking that of native tissues. An inverted version of this system has also been presented; Brassard and colleagues deposited HUVEC organoids, MSC aggregates, and intestinal organoids into matrigel-collagen suspension media before they were crosslinked. With the application of the correct growth factors, they were then able to demonstrate the self-organization of specific tissues to form connected vessel-like structures from the mm %–cm scale.^60^ They were also able to produce gradient structures by the co-extrusion of multiple organoid types, mimicking the stomach-intestine transition.^60^

Among different suspended printing techniques, all SM are rheologically similar in that they all exhibit minimal thixotropic behavior. A lack of standardization between labs, however, limits the comparisons that can be made between each technique. For example, in the techniques listed in Table II, different combinations of rheological tests were performed in each study. To enable better comparisons between suspension media and to identify the most appropriate media for a bioink, we suggest that at the minimum, the following should be reported: frequency sweeps, oscillatory strain and stress sweeps, thixotropic recovery, and in the case of particulate media, particle size analysis and volume fraction. Regarding suitability for individual bioinks, the minimum feature sizes achieved using each technique are reported using various inks and often do not include cells, limiting their translational relevance.

## VI. CONCLUSIONS AND FUTURE OUTLOOK

The term *bioprinting* was first defined in 2006 by Mironov *et al.*^145^ A decade later, after a number of previous iterations, *biofabrication* for TE was redefined by a consortium of international researchers including two of the previous trio.^146^ A short time later in 2018, *bioinks* was defined before a recent “roadmap” of the state of the field was presented.^13,147^ Efforts to standardize the field are ongoing, but with the rapid technological advances being made, this is a challenge. The most commonly cited limitation in extrusion bioprinting is appropriate bioink formulations for the production of functional tissues, but based on the last 5 years of research, the future looks bright. As the field continues to search for the optimal bioinks, standardizing the way that printability is determined is important to enable meaningful comparisons. Qualitative assessments by filament collapse and filament fusion tests are becoming more frequently used and may become standard in the literature. Testing parameters for quantitative rheological analysis, however, are still highly variable between labs and materials. The introduction of cells has a significant impact on bioink rheology with potential for both increased and decreased viscosities that will affect printability and shape resolution. While there exist limitations surrounding rheological analysis of cell-seeded bioinks, the cell-cell and cell-material chemical interactions must not be disregarded in bioink design and characterization.

The assessment of cell viability is fairly standardized by live/dead fluorescence imaging and metabolic assays consistently presented, as they have been in tissue engineering literature over the past four decades. Investigations into changing phenotypes during printing have shown that common EBP processes are gentle enough to prevent the induction of differentiation. A range of new bioinks have been developed in recent years, and while this review focused on those driven by rheological considerations, many groups are focusing on biological functionality. The use of decellularised ECM for bioinks ensures cell binding and enzyme-cleavable sites for tissue matrix regeneration and vascularization.^148,149^ Suspended printing holds a lot of promise, especially with the use of organoids to build constructs of physiologically relevant cell density with perfusable channels that will encourage vascular infiltration. While the printing of functional organs is still many years away, there have been many successes in printing functional pieces of tissue as well as bioprinting of *in vitro* models for drug screening. The commercialization of printing hardware, bioinks, and suspension media will make bioprinting accessible to both biologists and tissue engineers, which will help to drive the field into the next decade.

## Data Availability

The data that support the findings of this study are available from the corresponding author upon reasonable request.
